# Intraspinal clear cell meningioma without dural attachment: A case report and literature review

**DOI:** 10.1097/MD.0000000000025167

**Published:** 2021-03-19

**Authors:** Xiaolei Zhang, Peihai Zhang, James Jin Wang, Sheng Dong, Youtu Wu, Huifang Zhang, Guihuai Wang

**Affiliations:** Department of Neurosurgery, Beijing Tsinghua Changgung Hospital, School of Clinical Medicine, Tsinghua University, Beijing, China.

**Keywords:** case report, clear cell meningioma, meningioma, prognosis, recurrence rate

## Abstract

**Rationale:**

Clear cell meningioma (CCM) is one of the rarest but most aggressive forms of meningioma, with a tendency to occur at a high recurrence rate. Intraspinal CCM, especially the nondura-based type, is even rarer than the intracranial CCM.

**Patient concerns:**

We report a case of a 45-year-old woman who presented with a 1-month history of episodic pain in the lower back and in both thighs in the front side. Femoral nerve stretch tests were positive on both sides. Magnetic resonance imaging (MRI) demonstrated an intradural tumor at the L3 level, which was isointense on T1- and T2-weighted images (WI) and homogeneously enhanced on gadolinium-contrast T1 WI.

**Diagnoses:**

The space-occupying lesion was pathologically confirmed as CCM.

**Interventions:**

During surgery, we found that the tumor adhered to a nerve root, without dural attachment. The nerve root was partially removed to achieve complete resection.

**Outcomes:**

The pain disappeared after the operation. The 1 year follow-up MRI revealed no evidence of tumor recurrence or metastasis.

**Lessons:**

Nondura-based intraspinal CCM is easier to completely remove, and such complete removal should be achieved during the first operation. Although the recurrence rate of this particular type of meningioma appears to be lower than that of other types, close clinical and radiological follow-up is necessary.

## 1 Introduction

Clear cell meningioma (CCM), which was first described by Scheithauer^[1]^ in 1990, is one of the rarest forms of meningioma, accounting for only 0.2% of all cases. After its first description, CCM has been reported in the cerebellopontine angle, spinal/intradural (cervical, thoracic, and lumbar) locations, and supratentorial locations, with the lumbar region and cerebellopontine angle being the most common locations.

Histologically, CCM is composed of clear, glycogen-rich, polygonal cells, and the abundant glycogen explains the name “clear cell.”^[2,3]^ CCM differs from other varieties of meningioma in terms of its aggressive nature, high recurrence rate, and tendency to metastasize.^[4]^ According to the literature, the recurrence rate is 63.3% for intracranial CCM^[5,6]^ and 40% for intraspinal CCM.^[5,7,8]^ These clinical characteristics led the World Health Organization (WHO) to change the classification of CCM from grade I to grade II.^[9]^

Intraspinal CCM, especially the nondura-based type, is even rarer than the intracranial CCM. Herein, we report a case of intraspinal CCM without dural attachment and review the related literature. Our aim was to investigate the clinical characteristics, imaging features, treatment strategy, prognosis, and prognostic predictors of nondura-based intraspinal CCM. To the best of our knowledge, the number of patients included in our review is by far the largest in the literature.

## 2 Case report

### 2.1 Clinical history and physical examination

A 45-year-old woman presented with a 1-month history of episodic pain in her lower back and legs. Her pain was aggravated while changing posture, and it relieved after rest. The patient was treated only with medication (Loxoprofen) and the pain was partially relieved. No significant past trauma or medical or family history was noted. On physical examination, the pain was identified to occur in the lower back and in the front side of both thighs, without numbness. Femoral nerve stretch tests were positive on both sides, and worse on the left. The straight leg raising test was negative, and no positive results were found in muscle strength, sensation, deep tendon reflexes, Babinski sign, bladder function, or anal sphincter function.

### 2.2 Imaging findings

**Figure 1:**
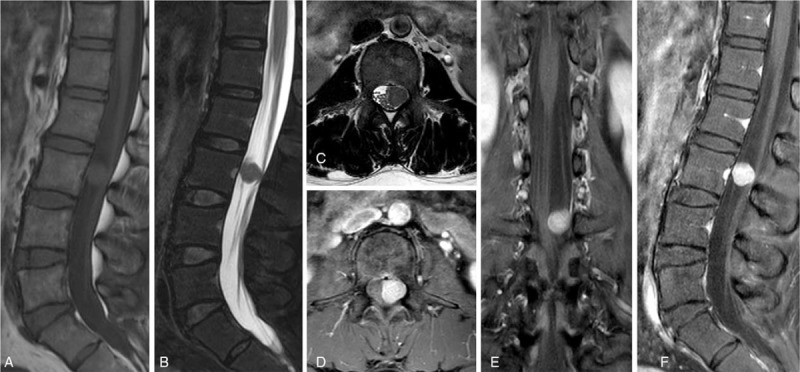
Preoperative spinal MRI showing a well-circumscribed enhancing mass at the L3 level. The mass, which was isointense on T1- and T2-weighted images (WI), homogeneously enhanced on gadolinium-contrast T1-WI, pushed the cauda equina to the right side, and occupied half the spinal canal volume. (A) Sagittal T1-WI; (B) sagittal T2-WI; (C) axial T2-WI; (D) axial contrast-enhanced image; (E) coronal contrast-enhanced image; (F) sagittal contrast-enhanced image. MRI = magnetic resonance imaging.

Lumbar spine magnetic resonance imaging (MRI) showed a well-demarcated, intradural oval lesion at the L3 level (Fig. 1). The lesion was isointense on T1- and T2-weighted images (WI). Gadolinium-contrast T1-WI revealed that the mass was homogeneously enhanced, pushed the cauda equina to the right side, and occupied half of the spinal canal volume. However, there was no evidence of the “dural tail sign.” The space-occupying lesion was differentially diagnosed as schwannoma or meningioma.

### 2.3 Operation

**Figure 2:**
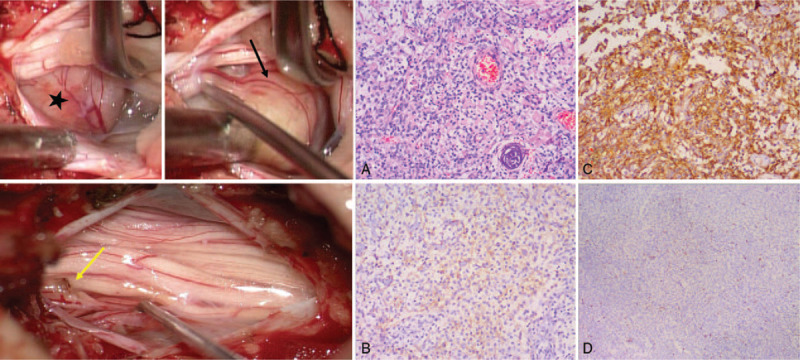
Intraoperative findings. A yellowish pink, oval, well-encapsulated tumor (marked with a pentagram) adhered to a nerve root (black arrow) without dural attachment. The nerve root was partially removed to achieve complete resection (the nerve stump is indicated with the yellow arrow). Histopathological findings revealed polygonal cells with clear glycogen-rich cytoplasm. (A) Hematoxylin and eosin stain, ×100. (B, C) The immunohistochemical stains showed a positive reaction with EMA (B) and vimentin (C). (D) The Ki-67 index was 20%. EMA = epithelial membrane antigen.

Under a small laminectomy and durotomy at the L3 level, we found a yellowish-pink, oval, well-encapsulated mass, which adhered to a nerve root without dural attachment (Fig. 2). Careful resection was performed using microsurgical techniques. However, the nerve root, which was tightly attached to the tumor, had to be partially removed to achieve complete resection.

### 2.4 Pathological results

The pathological results showed that the tumor was composed of polygonal cells with a clear glycogen-rich cytoplasm (Fig. 2). Immunohistochemically, the tumor cells were positive for vimentin and epithelial membrane antigen (EMA) but negative for periodic acid-Schiff staining (PAS) and S-100 protein. These findings were indicative of the diagnosis of clear cell meningioma. Moreover, the cells were positive for nuclear-associated antigen (Ki-67), with a labeling index of 10%, and for progesterone receptor (PR) expression.

### 2.5 Postoperative course

**Figure 3:**
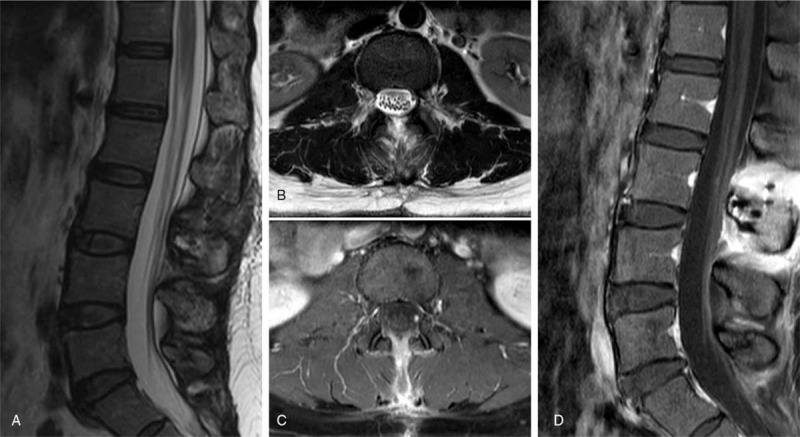
Postoperative spinal MRI (1-year follow-up) showed no evidence of tumor recurrence. (A) Sagittal T2-weighted image (WI); (B) axial T2-WI; (C) axial contrast-enhanced image; (D) sagittal contrast-enhanced image. MRI = magnetic resonance imaging.

The pain disappeared after the operation. No neurological deficits such as numbness, weakness, or sphincter dysfunction were found. The patient was very satisfied with the results of the operation and was discharged 7 days after surgery. At follow-up 1 month after surgery, the patient had involuntary twitching of the right thigh muscle, but the frequency was not high and did not affect her quality of life. The involuntary muscle twitching disappeared almost completely 10 months after surgery. One-year follow-up MRI (whole neuraxis) revealed no evidence of tumor recurrence or metastasis (Fig. 3).

Written informed consent was obtained from the patient for the publication of the case and all accompanying images, and the study design was approved by the ethics committee of authors’ institute.

## 3 Discussion

We present a case of intraspinal clear cell meningioma without dural attachment. To put the case into the context of current knowledge, and to attempt to draw some general conclusions about the prognosis and the treatment of this rare condition, we performed a review of similar cases reported in the literature. PubMed and Embase were searched with the keywords “nondura” and “intraspinal clear cell meningioma.” The references cited in the articles were also searched manually and carefully reviewed to identify potential additional studies. Detailed information about cases of nondura-based intraspinal CCM is shown in Table 1.

**Table 1:** Summary of the cases of nondura-based intraspinal clear cell meningioma reported in literature.

No.	Authors, year	Age, sex	Location	Root involvement, encapsulation	Resection	Removal of the nerve root	RT	Ki 67	Immunohistochemistry	Follow-up
1	Zorludemir S, et al^[5]^ 1995.	17, F	L4–5	Root (+), Capsule NA	Gross total resection	No	No	NA	NA	NED at 36 months
2	Holtzman RN, et al^[10]^ 1996.	32, M	L3–4	Root (+), Capsule (+)	Complete resection	No	No	NA	EMA (+), Vimentin (+), PR (+), ER (–)	NED at 1 month
3	Matsui H, et al^[11]^ 1998.	9, F	T11–12, L2, and L4–5. Multifocal.	Root (+), Capsule (+)	Complete resection	No	No	12%	NA	NED at 12 months
4	Dubois A, et al^[12]^ 1998.	10, F	L1–4	Root (+), Capsule (+)	Gross total resection	No	No	NA	Vimentin (+), NSE (+)	Recurrence in L1–2 at 6 months, followed by a second operation and RT.
5	Maxwell M, et al^[13]^ 1998.	31, F	L3	Root (+), Capsule NA	Complete resection	Yes	No	NA	Merlin (+)	NA
6	Carra S, et al^[14,15]^ 2001 and 2003.	22 months, M	T11-L4	Root (+), Capsule NA	Complete resection	NA	No	NA	NA	Recurrence in cerebellar vermis -C2 at 5 years, followed by total resection
7	Jallo GI, et al^[16]^ 2001.	8, F	L1–3	Root (+), Capsule (+)	Radical resection	NA	No	NA	NA	Recurrence in L3–5 at 6 months, followed by a second radical removal and RT.
8	Jallo GI, et al^[16]^ 2001.	22 months, F	C3–5	Root (+), Capsule (+)	Radical resection	NA	No	NA	NA	Recurrence at 5 months, followed by a second radical removal and RT; recurred again in the cerebellum 20 months later.
9	Florman J, et al^[17]^ 2001.	20, M	L4–5 and L5-S1. Multifocal.	Root (+), Capsule NA	Gross total resection	Yes	NA	NA	NA	NA
10	Cho CB, et al^[18]^ 2003.	17, F	S1–S2	Root (+), Capsule (+)	Complete resection	NA	NA	NA	Vimentin (+)	NA
11	Chen MH, et al^[19]^ 2004.	41, F	L4–5	Root (+), Capsule (+)	Total resection	NA	No	NA	EMA (+), Vimentin (+)	NED at 6 months
12	Payano M, et al^[3]^ 2004.	24, M	L3–4	Root (+), Capsule NA	Gross total resection	Yes	NA	<1%	Laminin (+)	NED at 61 months
13	Payano M, et al^[3]^ 2004.	19, F	L3–4	Root (+), Capsule NA	Complete resection	No	NA	<1%	Vimentin (+), EMA (+)	NED at 52 months
14	Oviedo A, et al^[6]^ 2005.	7, M	L2–3	Root (+), Capsule NA	Total resection	NA	No	10%	EMA (+), Vimentin (+), PR (+), ER (–)	NED at 12 months
15	Epstein NE, et al^[20]^ 2005.	41, F	L3–4	Root (+), Capsule NA	Gross total resection	Yes	No	2–3%	EMA (+)	NED at 6 months
16	Jia Y, et al^[21]^ 2005.	40, F	L1–2	Root (+), Capsule (+)	Complete resection	No	No	NA	EMA (+), Vimentin (+)	NED at 6 months
17	Nakajima H, et al^[22]^ 2009.	21, F	L2–3, and L4. Multifocal.	Root (+), Capsule (+)	Total resection	Yes	No	NA	EMA (+), Vimentin (+)	NED at 36 months
18	Ko JK, et al^[23]^ 2011.	34, F	L2–3	Root (+), Capsule (+)	Complete resection	No	No	NA	EMA: (+), Vimentin (+)	NED at 24 months
19	Kobayashi Y, et al^[24]^ 2013.	43, M	L1–3	Root (+), Capsule (+)	Complete resection	No	No	5%	EMA (+), PR (+), ER (–)	NED at 84 months
20	Schollenberg E, et al^[25]^ 2013.	54, M	L3–4	Root (+), Capsule: NA	NA	NA	No	NA	EMA (+)	NA
21	Li P, et al^[26]^ 2016.	7, F	L2–4	Root (+), Capsule NA	Total resection	NA	No	10%	NA	NED at 24 months
22	Kawasaki Y, et al^[27]^ 2018.	8, F	L3	Root (+), Capsule NA	Gross total resection	NA	No	26%	EMA (+); Vimentin (+)	NED at 24 months
23	This case	45, F	L3	Root (+), Capsule (+)	Complete resection	Yes	No	10%	Vimentin (+), EMA (+)	NED at 12 months

Capsule (+) = existence of a capsule as confirmed in the literature, EMA = epithelial membrane antigen, ER = estrogen receptor, NA = not available, NED = no evidence of disease, NSE = neuron-specific enolase, PAS = periodic acid-Schiff staining, PR = progesterone receptor, Root (+) = adhesion to the never root as confirmed in the literature, RT = radiotherapy.

### 3.1 Clinical and radiological features of nondura-based intraspinal CCM

Most spinal meningiomas have a dural attachment site, while nondura-based intraspinal CCMs are very rare. According to our review, 23 cases of nondura-based intraspinal CCM have been reported (Table 1). Patient age ranges from 1.8 to 54 years (mean, 23.1 years). The male/female ratio is 1:2.3. The lesion locations include lumbar segments (n = 18), thoracolumbar segments (n = 2), lumbosacral segments (n = 1), sacral segments (n = 1), and cervical segments (n = 1). The lumbar spine is thus the most common site of nondura-based intraspinal CCM.

The MRI features of nondura-based intraspinal CCM are similar to those of other types of meningioma: isointense on T1-WI, isointense or hyperintense on T2-WI, and strongly enhanced on gadolinium-contrast T1-WI.^[28]^ This type of intraspinal CCM is more common in the lumbar segments and is closely related to the cauda equina. In some cases, as in the one we reported, nerve root attachment could be clearly seen. The image-based diagnosis was often schwannoma or meningioma. Because of the lack of specificity in the imaging features, the gold standard for the diagnosis of nondura-based intraspinal CCM remains the pathological examination.

Regarding recurrence, 4 studies did not report follow-up and recurrence information and 4 patients experienced recurrence at various follow-up times; hence, the recurrence rate of nondura-based intraspinal CCM was 21.1% (4/19). The mean follow-up time was 19.3 months (range, 5–60) in 4 cases with recurrence and 26.4 months (range, 1–84) in 15 cases without recurrence. Although the extent of recurrence might be underestimated because the follow-up time was relatively short, the 21.1% recurrence rate was much lower than that previously reported (40% for intraspinal CCM and 63.3% for intracranial CCM), which is an interesting observation worthy of further analysis and discussion.

### 3.2 Predictors of CCM prognosis

It is generally believed that the extent of resection is the most important predictive factor of CCM prognosis, and total resection should be performed whenever possible to reduce the risk of recurrence.^[23,24,27]^ As for specific surgical techniques, Kawasaki et al^[24]^ suggested complete tumor removal and avoiding destroying the tumor capsule so as to reduce the chance of dissemination of the tumor cells during surgery. According to Nakajima et al,^[22]^ CCM should be removed totally together with the surrounding meninges (arachnoid and dura mater). If the tumor is tightly attached to the cauda equina (one of the most common areas of CCM), or the cauda equina is embedded within the tumor, then it is necessary to remove the pial membrane of the cauda equina, or even the cauda equina nerve root itself, to achieve complete resection.

Radiotherapy can be used as an adjunct therapy in patients whose tumor is difficult to remove completely such as some specific sites of intracranial CCM. Moreover, it has been suggested^[29]^ that adjuvant radiotherapy could be beneficial in patients with a high Ki-67 index to reduce the recurrence rate. Colen et al^[29]^ argued that radiation therapy is recommended in CCM, even when total resection has been achieved. However, patient 8 in our review^[16]^ underwent a second radical resection and radiotherapy after the tumor returned but the tumor returned again 20 months later. On the other hand, considering the lack of conclusive evidence and the potential harm inflicted by radiotherapy to the spinal cord (radiation-induced myelopathy) or other organs, especially in pediatric patients, we do not recommend adjunct radiotherapy for patients with CCM with total resection.

There is no consensus on whether the Ki-67 index can be used as a prognostic index for CCM. After reviewing 13 patients with CCM, Zorludemir et al^[5]^ found that the mean Ki-67 index of recurrent patients (13.3%; range, 3.3%–25.7%) was appreciably higher than that of non-recurrent patients (7.4%; range, 2.9%–17.2%), leading the authors to conclude that the Ki-67 index significantly differs between recurrent and non-recurrent CCM. However, cases of recurrence with a low Ki-67 index have been reported in the literature^[4]^ as well as cases with a high Ki-67 index without recurrence.^[12,14,27]^ Although there are few studies on Ki-67, it may have a predictive effect on CCM prognosis and is thus worthy of further research.

Other factors analyzed in the literature as prognostic predictors of CCM include PR-negativity and histological features. Previous analyses demonstrated that PR-negativity can be regarded as a prognostic factor in conventional meningioma,^[30,31]^ having, in particular, a strong predictive value for tumor recurrence (*P* < .0001).^[31]^ Regarding the relationship between PR-negativity and CCM prognosis, more data and research are necessary to reach a conclusion. Tong-tong et al^[2]^ suggested that CCM with anaplastic features represents an aggressive behavior that should be classified as WHO grade III. The extensive infiltrative growth pattern of CCM might hinder the achievement of complete microscopic resection.^[32]^ Whether there is a relationship between anaplastic features and CCM recurrence is unknown, and this still remains to be clarified because of the scarcity of relevant data.

### 3.3 Predictors of nondura-based intraspinal CCM prognosis

The patients in our review were divided into 2 groups according to the degree of tumor resection: group A (4 total resection cases and 8 complete resection cases) and group B (5 gross total resection cases and 2 radical resection cases). Recurrence occurred in 1 case in group A (1/12) and 3 cases in group B (3/7). It appears that the more thoroughly the tumor is removed, the lower the recurrence rate. When considering our review of nondura-based intraspinal CCM as a whole, we found that the recurrence rate of this CCM type was 21.1% (4/19), lower than other types of CCM. A possible reason for this difference is that surgical removal of this CCM type is more complete than the surgical removal of other types of CCM. Relevant supporting arguments are as follows: the anatomy of the spinal canal is relatively simple, allowing for a clearer surgical visualization. In our review, 78% (18/23) of the nondura-based intraspinal CCMs were located in the lumbar spine, making surgery relatively easier. In this series of cases, all tumors were attached to the nerve root rather than to the dura mater. This made it easier to remove the tumor completely without destroying its capsule. In some cases of this series, surgeons had to remove the nerve root that was tightly attached to the tumor, just as in our case. In such a situation, the tumor was removed together with the pial membrane of the nerve root, and the resection was therefore more thorough. Overall, nondura-based intraspinal CCM cases are easier to completely resect. Therefore, we believe that not only the extent of tumor resection but also the surgical techniques (avoiding the destruction of the tumor capsule and removing the surrounding meninges such as the arachnoid mater, the pial membrane of the nerve root, or even part of the nerve root) are the predictive factors of nondura-based intraspinal CCM prognosis.

None of the patients underwent radiotherapy after the first surgical resection, a finding that is consistent with most physicians’ opinion that radiotherapy is not recommended after total resection. Few studies are available to date regarding the predictive value of Ki-67, PR-negativity, and histological features. For example, in our review, specific Ki-67 values were reported for 9 patients only and for none of the 4 patients with recurrence. Therefore, further studies are needed to analyze the role of these factors.

### 3.4 Treatment strategy of nondura-based intraspinal CCM

Regarding the treatment strategies for nondura-based intraspinal CCM, the case we reported and those we reviewed lead us to the following recommendations: first, thanks to the anatomical features of the spinal canal, nondura-based intraspinal CCM should be totally removed during the first surgery whenever possible, and radiotherapy is not recommended after total resection, whether in children or in adults. Second, because of the potential aggressiveness and high recurrence rate of CCMs, we recommend close postoperative clinical and neuroradiological follow-up (with neuroimaging of the whole neuraxis every 6 months). Third, radiotherapy should be performed in cases of subtotal resection or of recurrence.

## 4 Limitations

Because of the rarity of the tumor, together with the short follow-up times, it is difficult to assess the recurrence rate of this tumor and to draw persuasive conclusions.

Therefore, we recommend that future studies on this rare tumor contain detailed information, including relevant clinical data before, during, and after surgery as well as imaging, pathological, immunological, and even genetic data. In this way, we will be able to achieve a better understanding of CCM.

## 5 Conclusion

Nondura-based CCM should be considered in patients with suspected meningioma in the lumbar spine. Nondura-based intraspinal CCM is easier to remove completely, and complete removal should be achieved during the first operation, even if it requires the sacrifice of a portion of the nerve root. Although the recurrence rate of this particular type of meningioma appears to be lower than that of other types, close clinical and radiological follow-up is recommended.

## Author contributions

**Conceptualization:** Xiaolei Zhang, Guihuai Wang.

**Data curation:** Xiaolei Zhang, Sheng Dong, Youtu Wu, Huifang Zhang.

**Formal analysis:** Youtu Wu.

**Methodology:** Sheng Dong.

**Supervision:** James Jin Wang, Guihuai Wang.

**Writing – original draft:** Xiaolei Zhang, Peihai Zhang.

**Writing – review & editing:** Xiaolei Zhang, James Jin Wang, Guihuai Wang.

## References

[1] Scheithauer BW. Tumors of the meninges: proposed modifications of the World Health Organization classification. Acta Neuropathol 1990;80:343–54. 2239146 10.1007/BF00307686

[2] Tong-tong W Li-juan B Zhi L. Clear cell meningioma with anaplastic features: case report and review of literature. Pathol Res Pract 2010;206:349–54. 19857933 10.1016/j.prp.2009.06.015

[3] Payano M Kondo Y Kashima K. Two cases of nondura-based clear cell meningioma of the cauda equina. APMIS 2004;112:141–7. 15056231 10.1111/j.1600-0463.2004.apm1120209.x

[4] Jain D Sharma MC Sarkar C. Clear cell meningioma, an uncommon variant of meningioma: a clinicopathologic study of nine cases. J Neurooncol 2007;81:315–21. 16955223 10.1007/s11060-006-9237-7

[5] Zorludemir S Scheithauer BW Hirose T. Clear cell meningioma. A clinicopathologic study of a potentially aggressive variant of meningioma. Am J Surg Pathol 1995;19:493–505. 7726360

[6] Oviedo A Pang D Zovickian J. Clear cell meningioma: case report and review of the literature. Pediatr Dev Pathol 2005;8:386–90. 16010490 10.1007/s10024-005-0119-3

[7] Dhall SS Tumialán LM Brat DJ. Spinal intradural clear cell meningioma following resection of a suprasellar clear cell meningioma. Case report and recommendations for management. J Neurosurg 2005;103:559–63. 16235691 10.3171/jns.2005.103.3.0559

[8] Li J Zhang S Wang Q. Spinal clear cell meningioma: clinical study with long-term follow-up in 12 patients. World Neurosurg 2019;122:e415–26. 30342264 10.1016/j.wneu.2018.10.064

[9] Zhou Q. [WHO classification of tumors of central nervous system (2007): an introduction]. Zhonghua Bing Li Xue Za Zhi 2008;37:5–7. 18509976

[10] Holtzman RN Jormark SC. Nondural-based lumbar clear cell meningioma. Case report. J Neurosurg 1996;84:264–6. 8592230 10.3171/jns.1996.84.2.0264

[11] Matsui H Kanamori M Abe Y. Multifocal clear cell meningioma in the spine: a case report. Neurosurg Rev 1998;21:171–3. 9795955 10.1007/BF02389326

[12] Dubois A Sévely A Boetto S. Clear-cell meningioma of the cauda equina. Neuroradiology 1998;40:743–7. 9860126 10.1007/s002340050676

[13] Maxwell M Shih SD Galanopoulos T. Familial meningioma: analysis of expression of neurofibromatosis 2 protein Merlin. Report of two cases. J Neurosurg 1998;88:562–9. 9488313 10.3171/jns.1998.88.3.0562

[14] Carrà S Drigo P Gardiman M. Clear-cell meningioma in a 22-month-old male: a case report and literature review. Pediatr Neurosurg 2001;34:264–7. 11423779 10.1159/000056035

[15] Carrà S Drigo P Gardiman M. Clear cell meningioma in a 22-month-old male: update after five years. Pediatr Neurosurg 2003;38:162–3. 12601242 10.1159/000068815

[16] Jallo GI Kothbauer KF Silvera VM. Intraspinal clear cell meningioma: diagnosis and management: report of two cases. Neurosurgery 2001;48:218–21. discussion 221-222. 11152351 10.1097/00006123-200101000-00042

[17] Florman J Khoshyomn S Tranmer B. Intraspinal clear cell meningioma: diagnosis and management--report of two cases. Neurosurgery 2001;49:481 doi: 10.1097/00006123-200108000-00055. PMID: 11504136. 10.1097/00006123-200108000-00055 11504136

[18] Cho CB Kim JK Cho KS. Clear cell meningioma of cauda equina without dural attachment. J Korean Neurosurg Soc 2003;34:584–5.

[19] Chen MH Chen SJ Lin SM. A lumbar clear cell meningioma with foraminal extension in a renal transplant recipient. J Clin Neurosci 2004;11:665–7. 15261248 10.1016/j.jocn.2003.10.024

[20] Epstein NE Drexler S Schneider J. Clear cell meningioma of the cauda equina in an adult: case report and literature review. J Spinal Disord Tech 2005;18:539–43. 16306847 10.1097/01.bsd.0000173314.98401.b5

[21] Jia Y Zhong DR Cui QC. Intraspinal clear cell meningioma: a case report. Chin Med J (Engl) 2005;118:348–9. 15740678

[22] Nakajima H Uchida K Kobayashi S. Microsurgical excision of multiple clear cell meningiomas of the cauda equina: a case report. Minim Invasive Neurosurg 2009;52:32–5. 19247902 10.1055/s-0028-1085455

[23] Ko JK Choi BK Cho WH. Non-dura based intaspinal clear cell meningioma. J Korean Neurosurg Soc 2011;49:71–4. 21494369 10.3340/jkns.2011.49.1.71 PMC3070901

[24] Kobayashi Y Nakamura M Tsuji O. Nondura-based clear cell meningioma of the cauda equina in an adult. J Orthop Sci 2013;18:861–5. 22437332 10.1007/s00776-012-0217-9

[25] Schollenberg E Easton AS. A case of clear cell meningioma with tyrosine-rich crystals. Int J Surg Pathol 2013;21:411–2. 23248340 10.1177/1066896912470165

[26] Li P Yang Z Wang Z. Clinical features of clear cell meningioma: a retrospective study of 36 cases among 10,529 patients in a single institution. Acta Neurochir (Wien) 2016;158:67–76. 26573513 10.1007/s00701-015-2635-x

[27] Kawasaki Y Uchida S Onishi K. Pediatric nondura-based clear cell meningioma of the cauda equina: case report and review of literature. Br J Neurosurg 2018;34:215–8. 29363346 10.1080/02688697.2018.1429565

[28] Lee W Chang KH Choe G. MR imaging features of clear-cell meningioma with diffuse leptomeningeal seeding. AJNR Am J Neuroradiol 2000;21:130–2. 10669237 PMC7976343

[29] Colen CB Rayes M McClendon J. Pediatric spinal clear cell meningioma. Case report. J Neurosurg Pediatr 2009;3:57–60. 19119906 10.3171/2008.10.17668

[30] Konstantinidou AE Korkolopoulou P Mahera H. Hormone receptors in non-malignant meningiomas correlate with apoptosis, cell proliferation and recurrence-free survival. Histopathology 2003;43:280–90. 12940781 10.1046/j.1365-2559.2003.01712.x

[31] Maiuri F De Caro MB Esposito F. Recurrences of meningiomas: predictive value of pathological features and hormonal and growth factors. J Neurooncol 2007;82:63–8. 17225937 10.1007/s11060-005-9078-9

[32] Yu KB Lim MK Kim HJ. Clear-cell meningioma: CT and MR imaging findings in two cases involving the spinal canal and cerebellopontine angle. Korean J Radiol 2002;3:125–9. 12087202 10.3348/kjr.2002.3.2.125 PMC2713835

